# Supplementary material for: Darwin Returns to the Galapagos: Genetic and Morphological Analyses Confirm the Presence of Tramea darwini at the Archipelago (Odonata, Libellulidae)
Source: Insects. 2020 Dec 31;12(1):21. doi: 10.3390/insects12010021 (PMC7823914; doi:10.3390/insects12010021)
Supplement: Supplementary file 1 [file insects-12-00021-s001.pdf]

insects MDPI

Supplementary Information File

Darwin returns to the Galapagos: genetic and morphological analyses confirm the presence of *Tramea darwini* at the archipelago (Odonata, Libellulidae)

M. Olalla Lorenzo-Carballa[1], Rosser W. Garrison[2], Andrea C. Encalada[3] & Adolfo Cordero-Rivera[1]

[1]*ECOEVO Group, EE Forestal, Campus Universitario A Xunqueira s/n, 36005, Pontevedra, SPAIN.*

[2]*California Department of Food & Agriculture 3294 Meadowview Road, Sacramento, CA 95832-1448, USA.*

[3]*Instituto BIOSFERA, Laboratorio de Ecología Acuática, Universidad San Francisco de Quito, ECUADOR.*

Author for correspondence: Adolfo Cordero-Rivera; e-mail address: adolfo.cordero@uvigo.gal

Supplementary Table S1: List of *Tramea* species included in the present study. For the specimens sequenced for this study (highlighted in bold), we list collection site, collection date, sex, collector/s data and GenBank accession numbers. Abbreviations for collectors are: AC-R - Adolfo Cordero-Rivera; AB - Alfredo Bolaños; MOL-C - M. Olalla Lorenzo-Carballa; RWG - Rosser W. Garrison; NvE - Natalia von Ellenrieder; RM - R. Mohabie; JK - Jens Kipping; PE - Paula Evans; AVE - Arthur V. Evans. n.a. indicates that a sequence could not be obtained for a particular specimen/locus.

Species	Voucher ID	Collection site	Coordinates	Sex	Collection date	Collector/s	GenBank accession Nos.			Reference
							COI	16S	ITS	
Tramea darwini	**ACR5165**	**Camino, Puerto Villamil, Isabela Island, Galapagos**	**0.9568 ºS 90.978 ºW**	♂	**1 August 2018**	**AC-R**	**MW246873**	**MW246903**	**MW246933**	**This study**
T. darwini	**ACR5171**	**Poza las Diablas, Puerto Villamil, Isabela Island, Galapagos**	**0.9571 ºS 90.978 ºW**	♀	**2 August 2018**	**AC-R**	**MW246874**	**MW246904**	**MW246934**	**This study**
T. darwini	**ACR5174**	**Poza las Diablas, Puerto Villamil, Isabela Island, Galapagos**	**0.9571 ºS 90.978 ºW**	♂	**2 August 2018**	**AC-R**	**MW246875**	**MW246905**	**MW246935**	**This study**
T. darwini	**ACR5175**	**Poza las Diablas, Puerto Villamil, Isabela Island, Galapagos**	**0.9571 ºS 90.978 ºW**	♀	**2 August 2018**	**AC-R**	**MW246876**	**MW246906**	**MW246936**	**This study**
T. darwini	**ACR5176**	**Poza las Diablas, Puerto Villamil, Isabela Island, Galapagos**	**0.9571 ºS 90.978 ºW**	♀	**2 August 2018**	**AC-R**	**MW246877**	**MW246907**	**MW246937**	**This study**
T. darwini	**ACR5177**	**Poza las Diablas, Puerto Villamil, Isabela Island, Galapagos**	**0.9571 ºS 90.978 ºW**	♂	**2 August 2018**	**AC-R**	**MW246878**	**MW246908**	**MW246938**	**This study**
T. darwini	**ACR5178**	**Cerro Mesa, Puerto Ayora, Santa Cruz Island, Galapagos**	**0.6433 ºS 90.2876 ºW**	♂	**7 August 2018**	**AC-R**	**MW246879**	**MW246909**	**MW246939**	**This study**
T. darwini	**ACR5179**	**Cerro Mesa, Puerto Ayora, Santa Cruz Island, Galapagos**	**0.6433 ºS 90.2876 ºW**	♀	**7 August 2018**	**AC-R**	**MW246880**	**MW246910**	**MW246940**	**This study**
T. darwini	**ACR5180**	**Cerro Mesa, Puerto Ayora, Santa Cruz Island, Galapagos**	**0.6433 ºS 90.2876 ºW**	♂	**7 August 2018**	**AC-R**	**MW246881**	**MW246911**	**MW246941**	**This study**

T. darwini	ACR5181	Cerro Mesa, Puerto Ayora, Santa Cruz Island, Galapagos	0.6433 °S 90.2876 °W	♂	7 August 2018	AC-R	MW246882	MW246912	MW246942	This study
T. darwini	ACR5182	Cerro Mesa, Puerto Ayora, Santa Cruz Island, Galapagos	0.6433 °S 90.2876 °W	♂	7 August 2018	AC-R	MW246883	MW246913	MW246943	This study
T. darwini	ACR5183	Cerro Mesa, Puerto Ayora, Santa Cruz Island, Galapagos	0.6433 °S 90.2876 °W	♀	7 August 2018	AC-R	MW246884	MW246914	MW246944	This study
T. darwini	F2MOLC	Road by Cucuve Eco-Hostal, San Cristobal Island, Galapagos	0.91 °S 89.589 °W	♀	1 August 2018	MOL-C	MW246885	MW246915	n.a.	This study
T. darwini	F3MOLC	Road by Cucuve Eco-Hostal, San Cristobal Island, Galapagos	0.91 °S 89.589 °W	♀	1 August 2018	MOL-C	MW246886	MW246916	MW246945	This study
T. darwini	F8MOLC	Finca Guadalupe, San Cristobal Island, Galapagos	0.927 °S 89.4862 °W	♀	2 August 2018	AB, MOL-C	MW246887	MW246917	MW246946	This study
T. darwini	M1MOLC	Road by Cucuve Eco-Hostal, San Cristobal Island, Galapagos	0.91 °S 89.589 °W	♂	1 August 2018	MOL-C	MW246888	MW246918	MW246947	This study
T. darwini	M2MOLC	Road by Cucuve Eco-Hostal, San Cristobal Island, Galapagos	0.91 °S 89.589 °W	♂	1 August 2018	MOL-C	MW246889	MW246919	MW246948	This study
T. darwini	M5MOLC	Finca Guadalupe, San Cristobal Island, Galapagos	0.927 °S 89.4862 °W	♂	2 August 2018	AB, MOL-C	MW246890	MW246920	MW246949	This study
T. darwini (= *calverti*)	RWG35312	Konawaruk watershed, Potaro-Siparuni Region, Guyana	5.315 °N 58.9067 °W	♀	19 September 2014	RWG, RM	MW246898	MW246928	MW246954	This study
T. darwini (= *calverti*)	RWG14805	Arroyo Yacui, Salta Province, Argentina	22.371 °S 63.7725 °W	♂	6 November 2006	RWG, NvE	MW246892	MW246922	MW246950	This study
T. cophysa	RWG42956	Roadside pool on route 2, Formosa Province, Argentina	26.049 °S 58.0667 °W	♀	5 November 2007	RWG, NvE	MW246902	MW246932	MW246955	This study
T. cophysa	RWG18540	Bañado La Estrella, Formosa Province, Argentina	24.459°S 60.3881 °W	♂	18 February 2008	RWG, NvE	MW246893	MW246923	MW246951	This study
T. cophysa	RWG27024	Dique el Tunal, Salta Province, Argentina	25.222°S 64.4753°W	♂	27 January 2012	NvE, RWG	MW246896	MW246926	MW246953	This study

T. abdominalis	**RWG14727**	**National Park El Rey, Salta Province, Argentina**	**24.709°S 64.640°W**	**♂**	**30 October 2006**	**RWG, NvE**	**MW246891**	**MW246921**	**n.a.**	**This study**
T. basilaris	**RWG36592**	**Shakawe-Sehitwa Nokaneng, Botswana,**	**19.511°S 22.124°E**	**♂**	**15 January 2009**	**JK**	**MW246899**	**MW246929**	**n.a.**	**This study**
T. basilaris	RF1190	-	-	-	-	-	AB709199	AB708255	AB707305	Futahasi 2011 [24]
T. basilaris	RF1191	-	-	-	-	-	AB709200	AB708256	AB707306	Futahasi 2011 [24]
T. basilaris	RF1192	-	-	-	-	-	AB709201	AB708257	AB707307	Futahasi 2011 [24]
T. binotata	**RWG26737**	**Pond by Ruta 2, Misiones Province, Argentina**	**25.851°S 54.557°W**	**♂**	**2 February 2012**	**RWG, NvE**	**MW246895**	**MW246925**	**MW246952**	**This study**
T. lacerata	**RWG42566**	**Harrison Lake National Fish Hatchery, Charles City Co., Virginia U.S.A.**	**37.336°N 77.188°W**	**♂**	**18 August 2018**	**RWG, NvE, AVE, PE**	**MW246901**	**MW246931**	**n.a.**	**This study**
T. lacerata	RF1682	-	-	-	-	-	AB709202	AB708258	AB707308	Futahasi 2011 [24]
T. virginia	**RWG33892**	**South China Agricultural University Guangdong Province, China**	**23.159°N 113.357°E**	**♂**	**12 June 2014**	**RWG**	**MW246897**	**MW246927**	**n.a.**	**This study**
T. virginia	RF964	-	-	-	-	-	AB709226	AB708282	AB707332	Futahasi 2011 [24]
T. virginia	RF1477	-	-	-	-	-	AB709227	AB708283	AB707333	Futahasi 2011 [24]
T. virginia	RF1478	-	-	-	-	-	AB709228	AB708284	AB707334	Futahasi 2011 [24]
T. carolina	**RWG24681**	**Harrison Lake National Fish Hatchery, Charles City Co., Virginia U.S.A.**	**25.851°S 54.557°W**	**♂**	**3 June 2011**	**RWG**	**MW246894**	**MW246924**	**n.a.**	**This study**
T. carolina	**RWG42564**	**Harrison Lake National Fish Hatchery, Charles City Co., Virginia U.S.A.**	**25.851°S 54.557°W**	**♂**	**18 August 2018**	**RWG, NvE, AVE & PE**	**MW246900**	**MW246930**	**n.a.**	**This study**

T. loewii	RF1751	-	-	-	-	-	AB709206	AB708262	AB707312	Futahasi 2011 [24]
T. loewii	RF1752	-	-	-	-	-	AB709207	AB708263	AB707313	Futahasi 2011 [24]
T. loewii	RF1753	-	-	-	-	-	AB709208	AB708264	AB707314	Futahasi 2011 [24]
T. propinqua	RF1324	-	-	-	-	-	AB709209	AB708265	AB707315	Futahasi 2011 [24]
T. transmarina	RF1553	-	-	-	-	-	AB709222	AB708278	AB707328	Futahasi 2011 [24]
T. transmarina	RF1583	-	-	-	-	-	AB709223	AB708279	AB707329	Futahasi 2011 [24]
T. transmarina	RF1754	-	-	-	-	-	AB709224	AB708280	AB707330	Futahasi 2011 [24]
Pantala flavescens	RF935	-	-	-	-	-	AB709104	AB708160	AB707210	Futahasi 2011 [24]

Supplementary Table S2: Primer combinations used to amplify mitochondrial (*COI* and *16S*) and nuclear (*ITS*) DNA of the *Tramea* species included in this study.

Locus	Forward primer (5′-3′)	Reverse primer (5′-3′)	Reference
Cytochrome oxidase I (*COI*)	Odo-LCO1490 TTTCTACWAACCAYAAAGATATTGG	Odo-HCO2198 TAAACTTCWGGRTGTCCAAARAATCA	Dijkstra et al 2014 [25]
	COI-S0 TACCAATTATAATTGGAGGATTYGG	COI-AS0 CTTCTGGATGTCCAAARAATCA	Futahasi 2011 [24]
Large ribosomal subunit (*16S*)	16S-H3080 CCGGTCTGAACTCAGATCACGT	16S-L2510 CGCCTGTTTATCAAAAACAT	Palumbi 1991 [23]
Internal Transcribed Spacer (*ITS*)	ITS-F0 GGAAAGATGGCCAAACTTGA	ITS-5.8S-AS2 CGTCGATGTTCATGTGTCCT	Futahasi 2011 [24]
	ITS-5.8S-S1 CGGTGGATCACTCGGCTCGT	ITS-28S-AS0 CCTCCGCTTATTAATATGCTTAAATTC	
		ITS-28S-AS6 CTTTTCCTCCGCTTATTAATATGCT	

Supplementary Table S3: Estimates of divergence over sequence pairs between the *Tramea* species included in this study (p-distances) as estimated by MEGA X for the mtDNA datasets. The number of base differences per site from averaging over all sequence pairs between groups are shown. Values above diagonal correspond to genetic distances estimated from the *COI* dataset, whereas values below diagonal correspond to distances estimated using the *16S* dataset.

	T. darwini	*T. calverti*	*T. cophysa*	*T. virginia*	*T. basilaris*	*T. propinqua*	*T. transmarina*	*T. loewii*	*T. lacerata*	*T. binotata*	*T. abdominalis*	*T. carolina*
T. darwini		0.004	0.033	0.072	0.086	0.071	0.074	0.070	0.117	0.115	0.076	0.091
T. calverti	0.000		0.036	0.075	0.088	0.074	0.076	0.073	0.119	0.116	0.078	0.093
T. cophysa	0.013	0.013		0.079	0.089	0.078	0.080	0.078	0.109	0.107	0.078	0.091
T. virginia	0.008	0.008	0.013		0.065	0.001	0.017	0.007	0.107	0.107	0.050	0.081
T. basilaris	0.010	0.010	0.019	0.007		0.066	0.066	0.066	0.130	0.128	0.075	0.096
T. propinqua	0.008	0.008	0.013	0.002	0.008		0.016	0.007	0.106	0.106	0.049	0.081
T. transmarina	0.009	0.009	0.014	0.003	0.009	0.005		0.018	0.105	0.104	0.050	0.081
T. loewii	0.008	0.008	0.013	0.000	0.006	0.002	0.003		0.104	0.104	0.050	0.079
T. lacerata	0.049	0.049	0.056	0.055	0.053	0.054	0.054	0.054		0.002	0.105	0.124
T. binotata	0.006	0.006	0.011	0.010	0.016	0.010	0.011	0.010	0.052		0.103	0.122
T. abdominalis	0.010	0.010	0.019	0.018	0.018	0.018	0.019	0.018	0.050	0.016		0.071
T. carolina	0.018	0.018	0.019	0.022	0.027	0.022	0.023	0.022	0.050	0.020	0.020	

Supplementary Table S4: Estimates of divergence over sequence pairs between the *Tramea* species included in this study (p-distances) as estimated by MEGA X for the nDNA dataset (*ITS*). The number of base differences per site from averaging over all sequence pairs between groups are shown.

	T. darwini	*T. calverti*	*T. cophysa*	*T. virginia*	*T. basilaris*	*T. propinqua*	*T. transmarina*	*T. loewii*	*T. lacerata*	*T. binotata*
T. darwini										
T. calverti	0.003									
T. cophysa	0.067	0.066								
T. virginia	0.033	0.031	0.070							
T. basilaris	0.038	0.037	0.079	0.015						
T. propinqua	0.033	0.031	0.070	0.006	0.014					
T. transmarina	0.042	0.041	0.077	0.010	0.023	0.013				
T. loewii	0.036	0.035	0.072	0.018	0.026	0.018	0.026			
T. lacerata	0.114	0.118	0.110	0.119	0.124	0.118	0.125	0.119		
T. binotata	0.020	0.018	0.068	0.027	0.036	0.027	0.036	0.030	0.115	

Supplementary Information Data S1: Results of species delimitation analyses for the *ITS* **(A)**, *16S* **(B)** and *COI* **(C)** *Tramea* datasets using ABGD (https://bioinfo.mnhn.fr/abi/public/abgd/abgdweb.html).

A:

ABGD Web results using JC69 Jukes-Cantor mesure of distance
Data: Ingroup_ITS_alignment.fasta

Jukes Cantor distance doneJC Partition 1 : found 25 groups (prior maximal distance P= 0.001000)
Partition 2 : found 8 groups (prior maximal distance P= 0.001668)
Partition 3 : found 7 groups (prior maximal distance P= 0.002783)
Partition 4 : found 7 groups (prior maximal distance P= 0.004642)
Partition 5 : found 3 groups (prior maximal distance P= 0.007743)
Partition 6 : found 1 groups (prior maximal distance P= 0.012915)

Histogram of distances

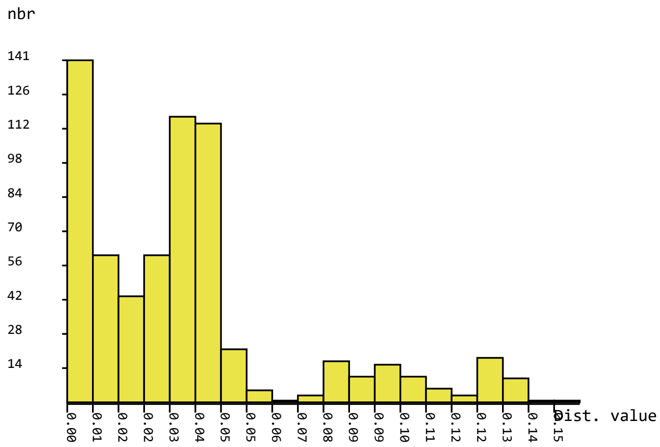

Ranked distances

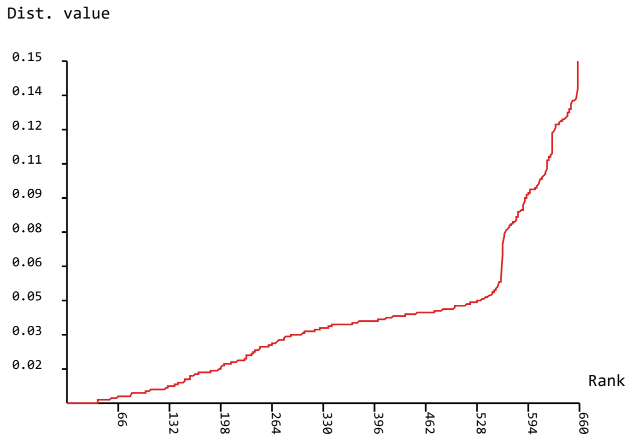

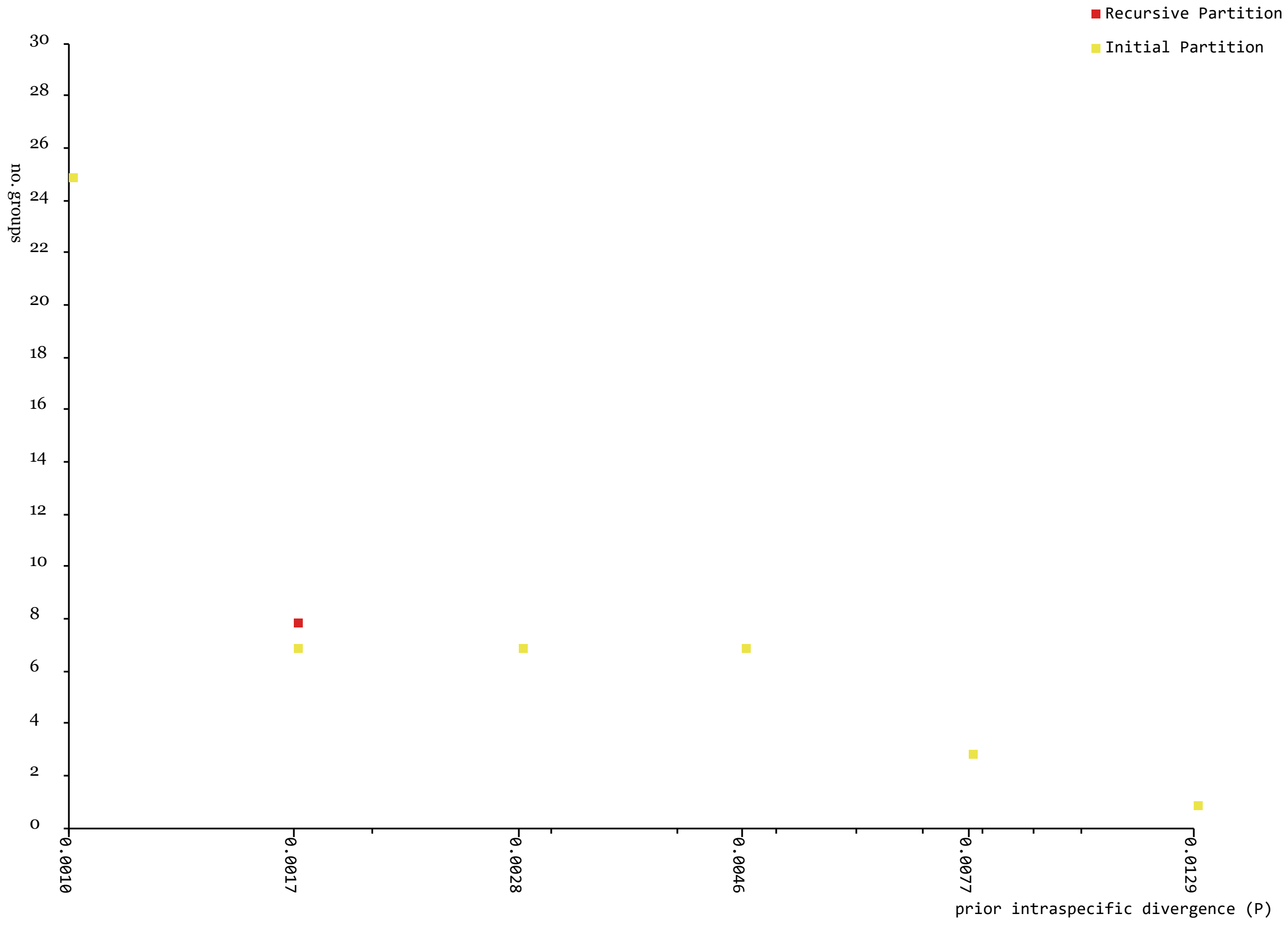

Groups identified by ABGD analysis for the ITS data:

Group[1] n: 19 ;id: ACR5165_Tramea_calverti ACR5171_Tramea_calverti ACR5174_Tramea_calverti ACR5175_Tramea_calverti ACR5176_Tramea_calverti ACR5177_Tramea_calverti ACR5178_Tramea_calverti ACR5179_Tramea_calverti ACR5180_Tramea_calverti ACR5181_Tramea_calverti ACR5182_Tramea_calverti ACR5183_Tramea_calverti F3-MOLC_Tramea_calverti F8-MOLC_Tramea_calverti M1-MOLC_Tramea_calverti M2-MOLC_Tramea_calverti M5-MOLC_Tramea_calverti RWG14805_Tramea_calverti RWG35312_Tramea_calverti
Group[2] n: 7 ;id: RF964_Tramea_virginia RF1324_Tramea_propinqua RF1477_Tramea_virginia RF1478_Tramea_virginia RF1553_Tramea_transmarina RF1583_Tramea_transmarina RF1754_Tramea_transmarina
Group[3] n: 3 ;id: RF1190_Tramea_basilaris RF1191_Tramea_basilaris RF1192_Tramea_basilaris
Group[4] n: 1 ;id: RF1682_Tramea_lacerata
Group[5] n: 3 ;id: RF1751_Tramea_loewii RF1752_Tramea_loewii RF1753_Tramea_loewii
Group[6] n: 3 ;id: RWG18540_Tramea_cophysa RWG27024_Tramea_cophysa RWG42956_Tramea_cophysa
Group[7] n: 1 ;id: RWG26737_Tramea_binotata

Group[1] n: 19 ;id: ACR5165_Tramea_calverti ACR5171_Tramea_calverti ACR5174_Tramea_calverti ACR5175_Tramea_calverti ACR5176_Tramea_calverti ACR5177_Tramea_calverti ACR5178_Tramea_calverti ACR5179_Tramea_calverti ACR5180_Tramea_calverti ACR5181_Tramea_calverti ACR5182_Tramea_calverti ACR5183_Tramea_calverti F3-MOLC_Tramea_calverti F8-MOLC_Tramea_calverti M1-MOLC_Tramea_calverti M2-MOLC_Tramea_calverti M5-MOLC_Tramea_calverti RWG14805_Tramea_calverti RWG35312_Tramea_calverti
Group[2] n: 4 ;id: RF964_Tramea_virginia RF1324_Tramea_propinqua RF1477_Tramea_virginia RF1478_Tramea_virginia
Group[3] n: 3 ;id: RF1190_Tramea_basilaris RF1191_Tramea_basilaris RF1192_Tramea_basilaris
Group[4] n: 1 ;id: RF1682_Tramea_lacerata
Group[5] n: 3 ;id: RF1751_Tramea_loewii RF1752_Tramea_loewii RF1753_Tramea_loewii
Group[6] n: 3 ;id: RWG18540_Tramea_cophysa RWG27024_Tramea_cophysa RWG42956_Tramea_cophysa
Group[7] n: 1 ;id: RWG26737_Tramea_binotata
Group[8] n: 3 ;id: RF1553_Tramea_transmarina RF1583_Tramea_transmarina RF1754_Tramea_transmarina

B:

ABGD Web results using JC69 Jukes-Cantor mesure of distance
Data: 16S_ingroup_alignment.fasta

Jukes Cantor distance doneJC Partition 1 : found 13 groups (prior maximal distance P= 0.001000)
Partition 2 : found 13 groups (prior maximal distance P= 0.001668)
Partition 3 : found 9 groups (prior maximal distance P= 0.002783)
Partition 4 : found 9 groups (prior maximal distance P= 0.004642)
Partition 5 : found 2 groups (prior maximal distance P= 0.007743)
Partition 6 : found 2 groups (prior maximal distance P= 0.012915)

Histogram of distances

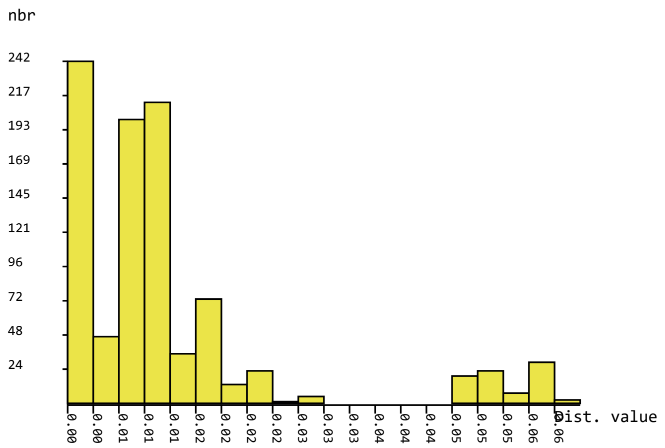

Ranked distances

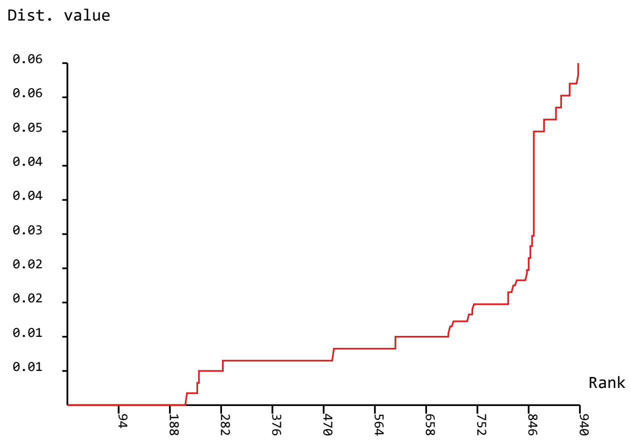

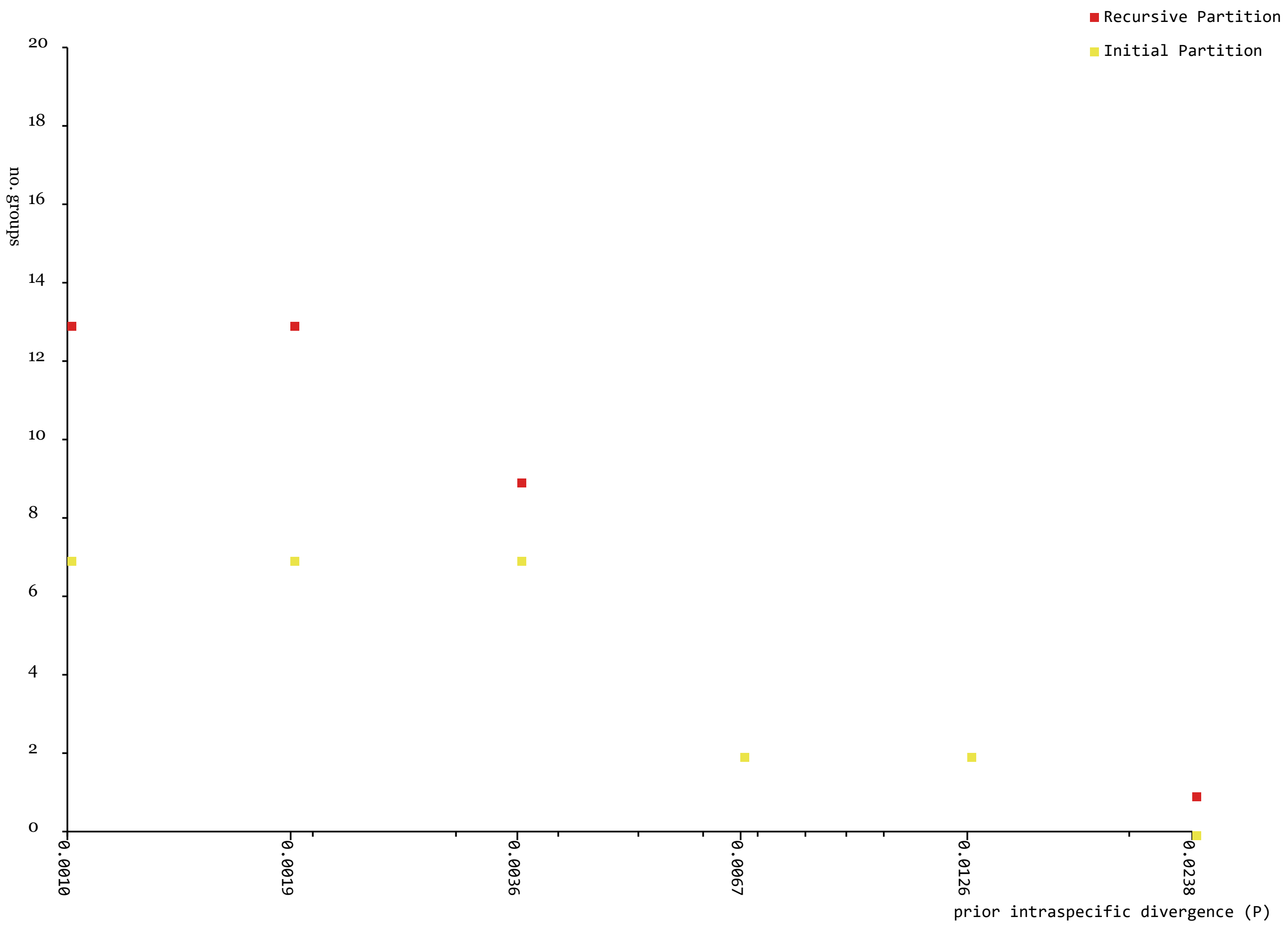

Groups identified by ABGD analysis for the 16S data:

Group[1] n: 21 ;id: ACR5165_Tramea_calverti ACR5171_Tramea_calverti ACR5174_Tramea_calverti ACR5175_Tramea_calverti ACR5176_Tramea_calverti ACR5177_Tramea_calverti ACR5178_Tramea_calverti ACR5179_Tramea_calverti ACR5180_Tramea_calverti ACR5181_Tramea_calverti ACR5182_Tramea_calverti ACR5183_Tramea_calverti F2-MOLC_Tramea_calverti F3-MOLC_Tramea_calverti F8-MOLC_Tramea_calverti M1-MOLC_Tramea_calverti M2-MOLC_Tramea_calverti M5-MOLC_Tramea_calverti RWG14805_Tramea_calverti RWG26737_Tramea_binotata RWG35312_Tramea_calverti
Group[2] n: 14 ;id: RF964_Tramea_virginia RF1190_Tramea_basilaris RF1191_Tramea_basilaris RF1192_Tramea_basilaris RF1324_Tramea_propinqua RF1477_Tramea_virginia RF1478_Tramea_virginia RF1583_Tramea_transmarina RF1751_Tramea_loewii RF1752_Tramea_loewii RF1753_Tramea_loewii RF1754_Tramea_transmarina RWG33892_Tramea_virginia RWG36592_Tramea_basilaris
Group[3] n: 1 ;id: RF1553_Tramea_transmarina
Group[4] n: 2 ;id: RF1682_Tramea_lacerata RWG42566_Tramea_lacerata
Group[5] n: 1 ;id: RWG14727_Tramea_abdominalis
Group[6] n: 3 ;id: RWG18540_Tramea_cophysa RWG27024_Tramea_cophysa RWG42956_Tramea_cophysa
Group[7] n: 2 ;id: RWG24681_Tramea_carolina RWG42564_Tramea_carolina

---

Group[1] n: 20 ;id: ACR5165_Tramea_calverti ACR5171_Tramea_calverti ACR5174_Tramea_calverti ACR5175_Tramea_calverti ACR5176_Tramea_calverti ACR5177_Tramea_calverti ACR5178_Tramea_calverti ACR5179_Tramea_calverti ACR5180_Tramea_calverti ACR5181_Tramea_calverti ACR5182_Tramea_calverti ACR5183_Tramea_calverti F2-MOLC_Tramea_calverti F3-MOLC_Tramea_calverti F8-MOLC_Tramea_calverti M1-MOLC_Tramea_calverti M2-MOLC_Tramea_calverti M5-MOLC_Tramea_calverti RWG14805_Tramea_calverti RWG35312_Tramea_calverti
Group[2] n: 10 ;id: RF964_Tramea_virginia RF1324_Tramea_propinqua RF1477_Tramea_virginia RF1478_Tramea_virginia RF1583_Tramea_transmarina RF1751_Tramea_loewii RF1752_Tramea_loewii RF1753_Tramea_loewii RF1754_Tramea_transmarina RWG33892_Tramea_virginia
Group[3] n: 1 ;id: RF1553_Tramea_transmarina
Group[4] n: 2 ;id: RF1682_Tramea_lacerata RWG42566_Tramea_lacerata
Group[5] n: 1 ;id: RWG14727_Tramea_abdominalis
Group[6] n: 3 ;id: RWG18540_Tramea_cophysa RWG27024_Tramea_cophysa RWG42956_Tramea_cophysa
Group[7] n: 2 ;id: RWG24681_Tramea_carolina RWG42564_Tramea_carolina
Group[8] n: 1 ;id: RWG26737_Tramea_binotata
Group[9] n: 4 ;id: RF1190_Tramea_basilaris RF1191_Tramea_basilaris RF1192_Tramea_basilaris RWG36592_Tramea_basilaris

C:

ABGD Web results using JC69 Jukes-Cantor mesure of distance
Data: COI_ingroup_alignment.fasta

Jukes Cantor distance doneJC Partition 1 : found 26 groups (prior maximal distance P= 0.001000)
Partition 2 : found 26 groups (prior maximal distance P= 0.001668)
Partition 3 : found 12 groups (prior maximal distance P= 0.002783)
Partition 4 : found 12 groups (prior maximal distance P= 0.004642)
Partition 5 : found 12 groups (prior maximal distance P= 0.007743)
Partition 6 : found 9 groups (prior maximal distance P= 0.012915)
Partition 7 : found 9 groups (prior maximal distance P= 0.021544)

Histogram of distances

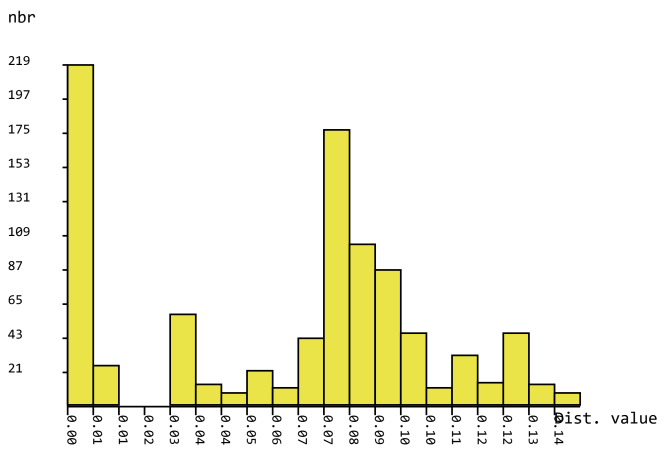

Ranked distances

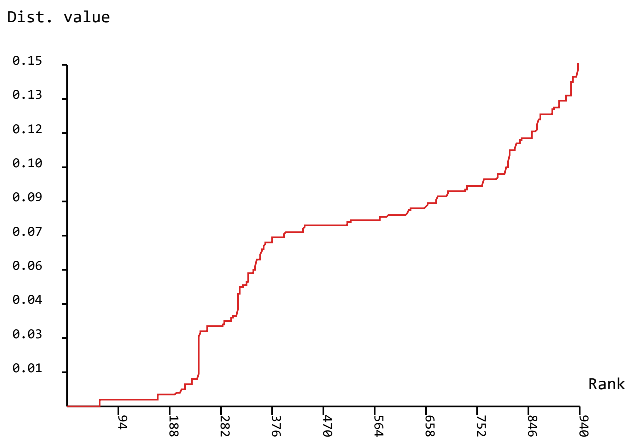

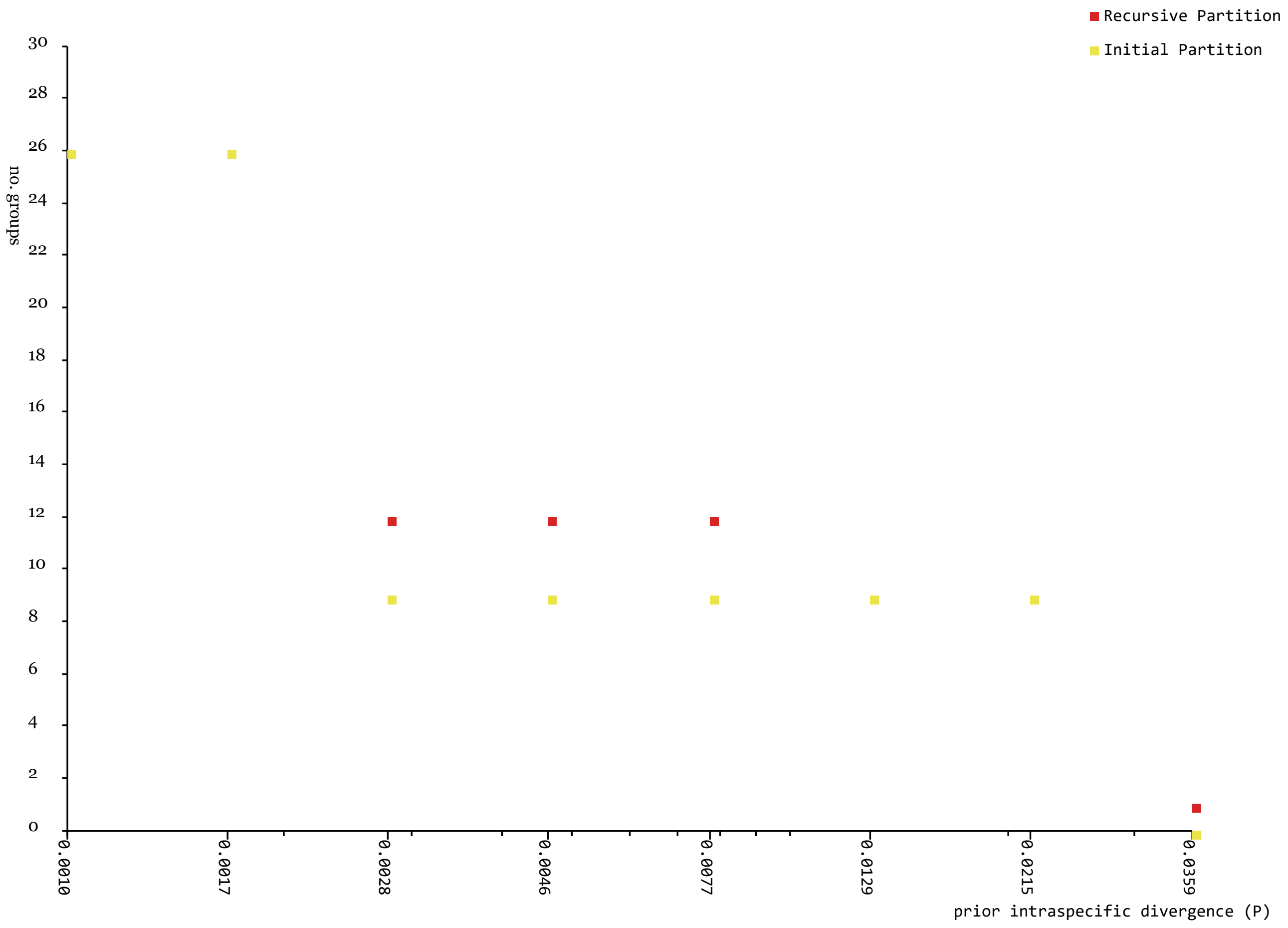

Groups identified by ABGD analysis for the COI data:

Group[1] n: 20 ;id: ACR5177_Tramea_calverti ACR5181_Tramea_calverti ACR5183_Tramea_calverti M5-MOLC_Tramea_calverti ACR5165_Tramea_calverti ACR5171_Tramea_calverti ACR5174_Tramea_calverti ACR5175_Tramea_calverti ACR5176_Tramea_calverti ACR5178_Tramea_calverti ACR5179_Tramea_calverti ACR5180_Tramea_calverti ACR5182_Tramea_calverti F2-MOLC_Tramea_calverti F3-MOLC_Tramea_calverti F8-MOLC_Tramea_calverti M1-MOLC_Tramea_calverti M2-MOLC_Tramea_calverti RWG14805_Tramea_calverti RWG35312_Tramea_calverti
Group[2] n: 10 ;id: RF964_Tramea_virginia RF1324_Tramea_propinqua RF1477_Tramea_virginia RF1478_Tramea_virginia RF1583_Tramea_transmarina RF1751_Tramea_loewii RF1752_Tramea_loewii RF1753_Tramea_loewii RF1754_Tramea_transmarina RWG33892_Tramea_virginia
Group[3] n: 3 ;id: RF1190_Tramea_basilaris RF1191_Tramea_basilaris RF1192_Tramea_basilaris
Group[4] n: 1 ;id: RF1553_Tramea_transmarina
Group[5] n: 3 ;id: RF1682_Tramea_lacerata RWG26737_Tramea_binotata RWG42566_Tramea_lacerata
Group[6] n: 1 ;id: RWG14727_Tramea_abdominalis
Group[7] n: 3 ;id: RWG18540_Tramea_cophysa RWG27024_Tramea_cophysa RWG42956_Tramea_cophysa
Group[8] n: 2 ;id: RWG24681_Tramea_carolina RWG42564_Tramea_carolina
Group[9] n: 1 ;id: RWG36592_Tramea_basilaris

Group[1] n: 20 ;id: ACR5177_Tramea_calverti ACR5181_Tramea_calverti ACR5183_Tramea_calverti M5-MOLC_Tramea_calverti ACR5165_Tramea_calverti ACR5171_Tramea_calverti ACR5174_Tramea_calverti ACR5175_Tramea_calverti ACR5176_Tramea_calverti ACR5178_Tramea_calverti ACR5179_Tramea_calverti ACR5180_Tramea_calverti ACR5182_Tramea_calverti F2-MOLC_Tramea_calverti F3-MOLC_Tramea_calverti F8-MOLC_Tramea_calverti M1-MOLC_Tramea_calverti M2-MOLC_Tramea_calverti RWG14805_Tramea_calverti RWG35312_Tramea_calverti
Group[2] n: 7 ;id: RF964_Tramea_virginia RF1324_Tramea_propinqua RF1477_Tramea_virginia RF1478_Tramea_virginia RF1752_Tramea_loewii RF1754_Tramea_transmarina RWG33892_Tramea_virginia
Group[3] n: 3 ;id: RF1190_Tramea_basilaris RF1191_Tramea_basilaris RF1192_Tramea_basilaris
Group[4] n: 1 ;id: RF1553_Tramea_transmarina
Group[5] n: 3 ;id: RF1682_Tramea_lacerata RWG26737_Tramea_binotata RWG42566_Tramea_lacerata
Group[6] n: 1 ;id: RWG14727_Tramea_abdominalis
Group[7] n: 3 ;id: RWG18540_Tramea_cophysa RWG27024_Tramea_cophysa RWG42956_Tramea_cophysa
Group[8] n: 2 ;id: RWG24681_Tramea_carolina RWG42564_Tramea_carolina
Group[9] n: 1 ;id: RWG36592_Tramea_basilaris
Group[10] n: 1 ;id: RF1583_Tramea_transmarina
Group[11] n: 1 ;id: RF1751_Tramea_loewii
Group[12] n: 1 ;id: RF1753_Tramea_loewii
